# On the Role of Atmospheric Weathering on Paint Dust Aerosol Generated by Mechanical Abrasion of TiO_2_ Containing Paints

**DOI:** 10.3390/ijerph19031265

**Published:** 2022-01-24

**Authors:** Adam W. Nored, Jacob S. Shedd, Marie-Cecile G. Chalbot, Ilias G. Kavouras

**Affiliations:** 1 Interdisciplinary Engineering Program, School of Engineering, University of Alabama at Birmingham, 1075 13th St. S, Birmingham, AL 35205, USA; anored@gmail.com; 2 Department of Environmental Health Sciences, Ryals School of Public Health, University of Alabama at Birmingham, 1665 University Blvd, Birmingham, AL 35233, USA; jshedd91@uab.edu (J.S.S.); ilias.kavouras@sph.cuny.edu (I.G.K.); 3 Department of Biological Sciences, School of Arts and Sciences, New York City College of Technology, 285 Jay St., New York, NY 11201, USA; 4 Department of Environmental, Occupational and Geospatial Health Sciences, CUNY Graduate School of Public Health and Health Policy, 55 W 125th Street, New York, NY 10024, USA

**Keywords:** Engineered nanomaterials, weathering, air pollutants, meteorology, acidity, titanium dioxide

## Abstract

In recent years, the introduction and use of new nanomaterials in construction has increased at a rapid rate. Exterior surface paints have been a product that have had these nanomaterials added to them. In this study, the effects of natural weathering and exposure to atmospheric agents was examined to determine the detrimental effects on outdoor paint that has been created with nanomaterials. Data collected over the course of the yearlong study indicate that the nanoparticles of the titanium dioxide were eliminated rapidly. Further testing indicated that various elements of weathering were affecting the physical integrity of the paint. The weathering agents that appeared to have the greatest effect on the samples were acid deposition and total precipitation. There was a strong association between carbon monoxide and the effects on the panels. These results can lead to new plans for assessments involving the synergistic effects of all weathering agents.

## 1. Introduction

Engineered nanomaterials (ENMs) have been integrated into construction materials including paints [1]. These nano-enabled products (NEPs) are commercially desirable due to their improved structural integrity, thermal conductivity, fire prevention, and self-cleaning features as compared to conventional products, and, as such, they are extensively used in commercially available consumer products [2,3,4,5]. Despite these advantages, most commercially available NEPs are not properly identified as containing nanomaterials. Moreover, knowledge about their health and environmental effects is scarce, particularly throughout the lifecycle of the product. For paint, it includes preparation and application on indoor and outdoor surfaces, environmental weathering and aging, and final removal, typically by mechanical abrasion. Many ENMs, such as carbon nanotubes, silicon dioxide (SiO_2_), titanium dioxide (TiO_2_), and copper oxide (CuO) have been shown to be harmful to humans, with pristine nanoparticles translocating from the lungs into the circulatory, lymphatic, and nervous systems [6,7,8,9,10].

Exposures of painters and other construction workers are most likely to occur at the early and last stages of the paint lifecycle. The sanding dust of walls and wood coated with nano-containing products was dominated by particles in the 100–300 nm size range [11]. TiO_2_ and Ag nanoparticles were found in 80% of the collected paint dust particles [12]. The size of TiO_2_-containing paint dust and the abundance of nano-sized TiO_2_ agglomerates was related to the sandpaper grit size [13]. As a result, there is potential for occupational exposures to nanoparticles in paints. This may increase the probability of various diseases that involve the lungs, as painters already have consistently higher lung cancer mortality than other construction workers [14]. Additionally, the probability of a painter developing chronic obstructive pulmonary disease (COPD) is the second highest among all construction workers, following roofers [15].

Exposure to environmental conditions including weather and chemicals affects the integrity and stability of paint over time, leading to deterioration, breaks, and the release of chemical species. Kaegi et al. [16] determined that leachate from a two-year-old weather-exposed façade had significantly less ENMs than a freshly coated façade. Silver ENMs leaching showed that the first two months of exposure resulted in 30% of total ENM loss [17]. Atmospheric pollution can also be detrimental to coatings over time. Acid deposition is known to significantly deteriorate the integrity of outdoor surfaces [18,19]. TiO_2_, the most widely used pigment in paints, reacts with acids through photocatalysis, potentially leading to the destruction of the coating binders in the outer paint film [20,21]. This may be enhanced by the interaction of anatase nano TiO_2_ and UV light [22]. As a result, paint chemicals and ENMs may be released into the environment and enter the human body through a variety of pathways including hand-to-mouth for toddlers, the primary concern for Pb-based paint, and through the food chain (i.e., bioaccumulation) [23,24,25].

In the present work, we aim to evaluate the effect of environmental weathering on the release of TiO_2_-containing paint dust by mechanical abrasion. The premise behind this study is that environmental weathering may deteriorate the integrity of paint over time, affecting both the availability and ease of generation of TiO_2_-containing paint dust by mechanical abrasion. Using a previously developed methodological framework [13], the current work sought to realistically simulate the generation of TiO_2_-containing paint dust by mechanical abrasion on weathered wood panels for a year.

## 2. Materials and Methods

### 2.1. Atmospheric Weathering Experiment

To evaluate the role of environmental weathering on paint dust generated by mechanical abrasion, wood panels painted with TiO_2_-containing paint were exposed to weather conditions and air contaminants for regular intervals up to a year. More specifically, both sides of twelve (12) wood panels were prepared, conditioned, and coated as previously described [13]. Briefly, the two sides of the wood panels (61.25 cm [L] × 28.75 cm [W]) were painted with two layers of a commercially available water-based (49.6% *w*/*w*) latex paint and primer formulation containing TiO_2_ (3.2% *w*/*w*). The application was done manually using a 5-cm brush. The panels were conditioned at 20 °C and 30% RH for 48 h between coatings and for 24 h before exposure. All panels were placed on the roof of a 6-floor university building with restricted access and at least 10 m away from major air intake and outlets to prevent unwanted contamination and interference (latitude: 33.502001; longitude: −86.80382 (Datum: WGS84)) from 1 February 2017 to 31 January 2018. Every fifteen days, the panels were rotated to directly expose both sides to environmental conditions. A panel was randomly selected every 30 days for mechanical abrasion.

Meteorological conditions and air pollution concentrations measured at the US Environmental Protection Agency NCore site in Birmingham, Alabama (EPA AIRS ID: 01-073-0023) (latitude: 33.553056; longitude: −86.815000 (Datum: WGS84); Elevation 177 m above sea level) for 2017 and 2018 were retrieved from the US Environmental Protection Agency’s Air Quality System (AQS). The measured meteorological conditions included the hourly temperature (in °F), percent of relative humidity, barometric pressure (in mbar), total precipitation (in inches of water), resultant wind speed (in knots), and solar radiation (in Langley per minute). Gaseous pollutant criteria included hourly CO (in ppm), O_3_ (in ppm), NO_x_ (in ppb), and SO_2_ (in ppb). The PM_10_ and PM_2.5_ mass (in μg/m^3^) were measured daily, and PM_2.5_ speciation data were obtained at a frequency of once every three days. More specific chemical components including the total sulfate (SO_4_^2−^), nitrate (NO_3_^−^), ammonium bisulfate ((NH_4_)_2_SO_4_), and ammonium nitrate (NH_4_NO_3_) concentrations were obtained.

### 2.2. Paint Dust Generation and Characterization

The Coatings Aerosol Resuspension System (CARES) was used to generate TiO_2_ containing paint dust using an orbital sander and 120 grit size sandpaper in a polyvinylchloride glove box chamber (115 cm × 60 cm × 60 cm) (Lancs Industries, Kirkland, WA, USA) [13]. A wide particle spectrometer (WPS) (Model 1000XP, MSP Corp., Shoreview, MN, USA) and a condensation particle counter (CPC) (Model 3771, TSI Inc., Shoreview, MN, USA) were used to measure the particle number concentration and size distribution. Paint dust samples were collected on 13-mm cellulose filters and 47-mm Teflon filters (Pall Corp., Port Washington, NY, USA) at a 1.0 and 5.0 L/min flow rate, respectively, for chemical and imaging analysis, respectively. Chemical analysis was done by attenuated total reflectance Fourier-transform infrared spectrometer (ATR-FTIR; ALPHA II Platinum ATR, Bruker corp., Billerica, MA, USA) with a single reflection diamond ATR (range: 400–4000 cm^−1^, with a resolution of 2 cm^−1^). Morphological analysis was performed by scanning electron microscope (SEM; 650 FEG, Eindhoven, The Netherlands). The SEM was used at a low pressure setting of 0.53 torr, working distance of 10 mm, and accelerating voltage of 10 kV. Samples were examined at 5000× and 10,000× magnifications. The elemental composition was determined using the integrated energy-dispersive X-ray (EDX) at 5000× magnification.

### 2.3. Data Analysis

The count median diameter (CMD), geometric standard deviation (GSD), and PM_10_, PM_2.5_, and PM_1_ mass concentration of paint dust were computed using the WPS data, as previously described [13]. Daily descriptive statistics or meteorological and air pollution variables were computed from hourly measurements. Subsequently, daily estimates were further aggregated to compute the cumulative exposure conditions for each wood panel over the 48-week period. Aerosol acidity (in mol/m^3^) was computed as the sum of the molar concentrations of free SO_4_^2−^ (difference of total SO_4_^2−^ and neutralized (NH_4_)_2_SO_4_) and free NO_3_^−^ (difference of total NO_3_^−^ and neutralized NH_4_NO_3_). The sum of gaseous SO_2_ and NO_x_ molar concentrations (in mol/m^3^) was defined as the gas acidity. Ordinary least squares regression analyses of CMD, GSD, PM_2.5_, and PM_1_ mass as well as source contributions were used to determine the monthly trends. A multivariate least squares linear regression analysis was used to estimate the synergistic effect of weathering conditions on paint dust levels. The following weather conditions were included in the analysis: dew point (computed from the ambient temperature, percent of relative humidity, and barometric pressure), molar CO and O_3_ concentrations, precipitation, wind speed, and aerosol and gas acidity, allowing for *df* = 4 (degrees of freedom of the model). The percent of relative effect of each variable was calculated as the product of the regression coefficient and cumulative exposure for each variable to the total effect of all variables. The intercept is interpreted as being the effect associated with other weathering variables not included in this study such as highly reactive radicals. All analyses were done using SPSS (Version 27; IBM Analytics, Armonk, NY, USA) and Origin Pro (Version 9.1; OriginLab, Northampton, MA, USA).

## 3. Results and Discussion

### 3.1. Paint Dust Concentrations and Trends

Table 1 presents the mean (±3× standard error) and monthly trend of the CMD, GSD, and reconstructed PM_10_, PM_2.5_, and PM_1_ mass concentrations. The CMD of paint dust varied from 39 ± 10 nm to 98 ± 29 nm with a monthly increase of 2.2 ± 1.2 nm/month. The GSDs of paint dust particles decreased from 3.9 ± 0.7 to 2.3 ± 0.1, at a rate of 0.12 ± 0.02 nm/month, suggesting a narrow size range of paint dust particles after weathering. The CMD and GSD of paint dust generated after one month of environmental weathering were somewhat different than those computed for paint dust generated from freshly painted wood panels using the same protocol (CMD of 39.4 ± 2.7 nm; GSD of 2.2 ± 0.1), albeit similar levels of particle mass (PM_10_, PM_2.5_, and PM_1_) levels were generated.

This may be suggestive of weathering processes affecting the physical integrity of paints and therefore the number and size of generated paint dust, but with no significant changes in the availability of paint. The PM_10_, PM_2.5_, and PM_1_ paint dust concentrations varied month by month; however, an overall decline was observed for all three fractions, from −123 ± 35 μg/m^3^/month for PM_10_ to −5 ± 3 μg/m^3^/month for PM_2.5_ and −1 ± 0.8 μg/m^3^/month for PM_1_. This trend indicated that weathering may reduce the quantity of paint available for mechanical abrasion through physical and chemical deterioration and break-up.

### 3.2. Chemical and Morphological Analysis

**Figure 1:**
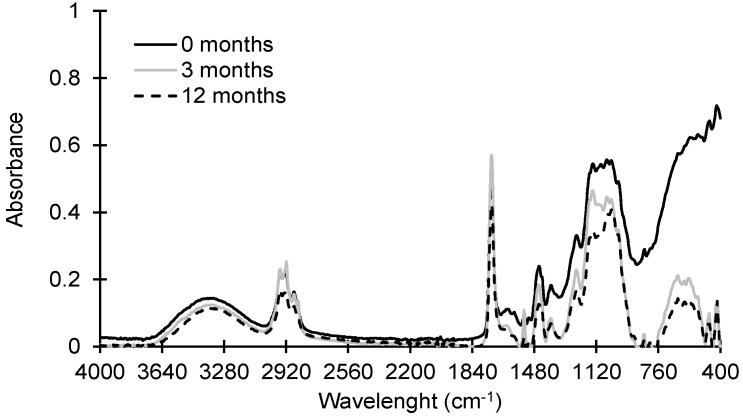
ATR-FTIR spectra of paint dust generated by sanding using P120 sandpaper after 0, 3, and 12 months of environmental weathering.

The ATR-FTIR absorbance spectra of paint dust at 0, 3, and 12 months are depicted in Figure 1. All paint dust samples retained the absorption bands at 1500–3500 cm^−1^ from wood cellulosic signatures and paint organic polymers [13,26]. However, there was a decline in the absorption bands of 1240–750 cm^−1^, attributed to the stretching of =C–H and C–O groups and bending vibration of the aromatic C–H groups.

Olefinic, aromatic, and hydroxyl functional groups may be more susceptible to reactions by atmospheric oxidants, leading to the rearrangement and break-up of the aliphatic chain in organic polymers and/or hydrogen bonds between polymers [27,28]. The abundance of broad bands in the 400–650 cm^−1^ regions previously assigned to TiO_2_ was observed to substantially decline following three months of environmental weathering, indicating the rapid elimination of TiO_2_ materials from painted surfaces due to weathering.

**Figure 2:**
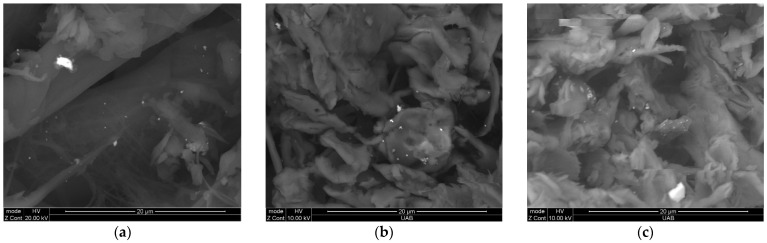
Backscattering SEM image of paint dust generated by sanding using P120 sandpaper after (**a**) 0, (**b**) 3, and (**c**) 12 months of environmental weathering.

Figure 2 depicts the SEM backscattering images of paint dust at months (a) 0, (b) 3, and (c) 12. Released paint dust particles contained TiO_2_ nanomaterials of various shapes (spherical, amorphous) in the nano-size and submicron size range at 0 months. Both the size and concentration of TiO_2_ paint dust agglomerates declined following weathering, indicating that most of the TiO_2_ may be inadvertently released into the environment; however, the remaining TiO_2_ may be released in the nano-size range by mechanical abrasion.

### 3.3. Weathering

**Figure 3:**
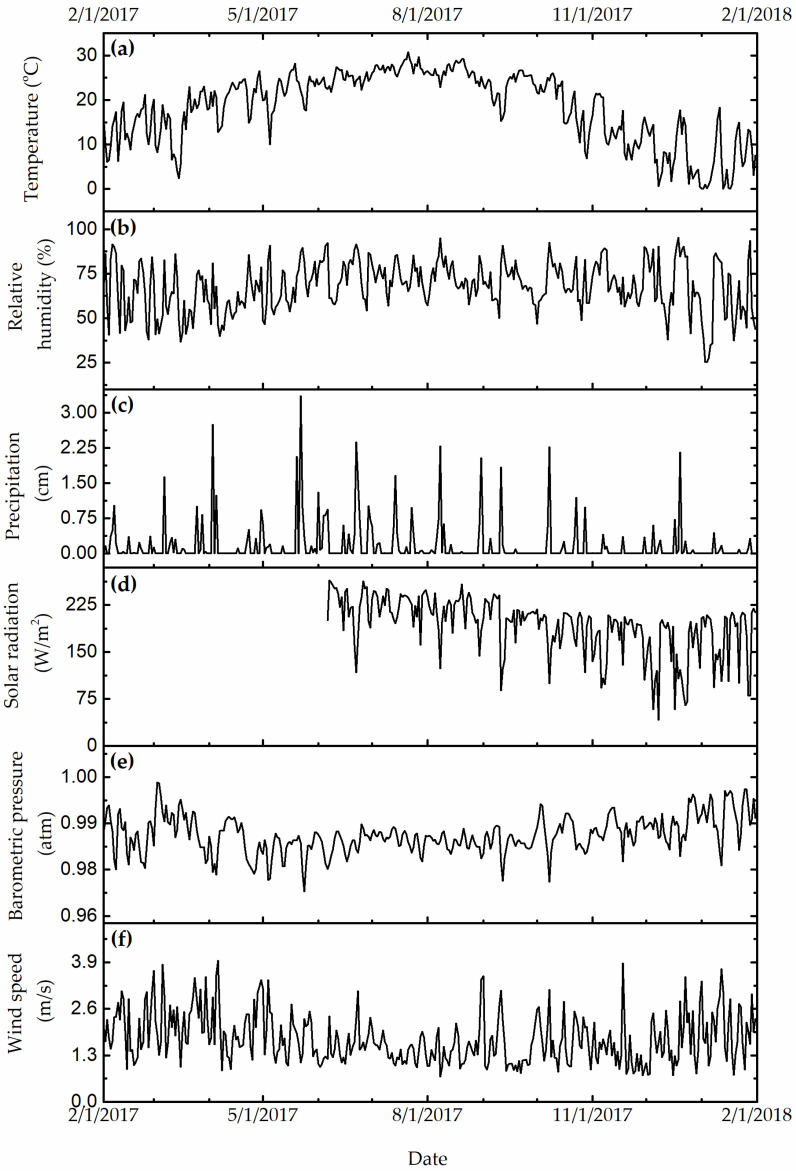
(**a**, °C) Daily temperature, (**b**, %) relative humidity, (**c**, cm of H_2_O) precipitation, (**d**, W/m^2^) solar radiation, (**e**, in mbar) barometric pressure, and (**f**, m/s) resultant wind speed.

**Figure 4:**
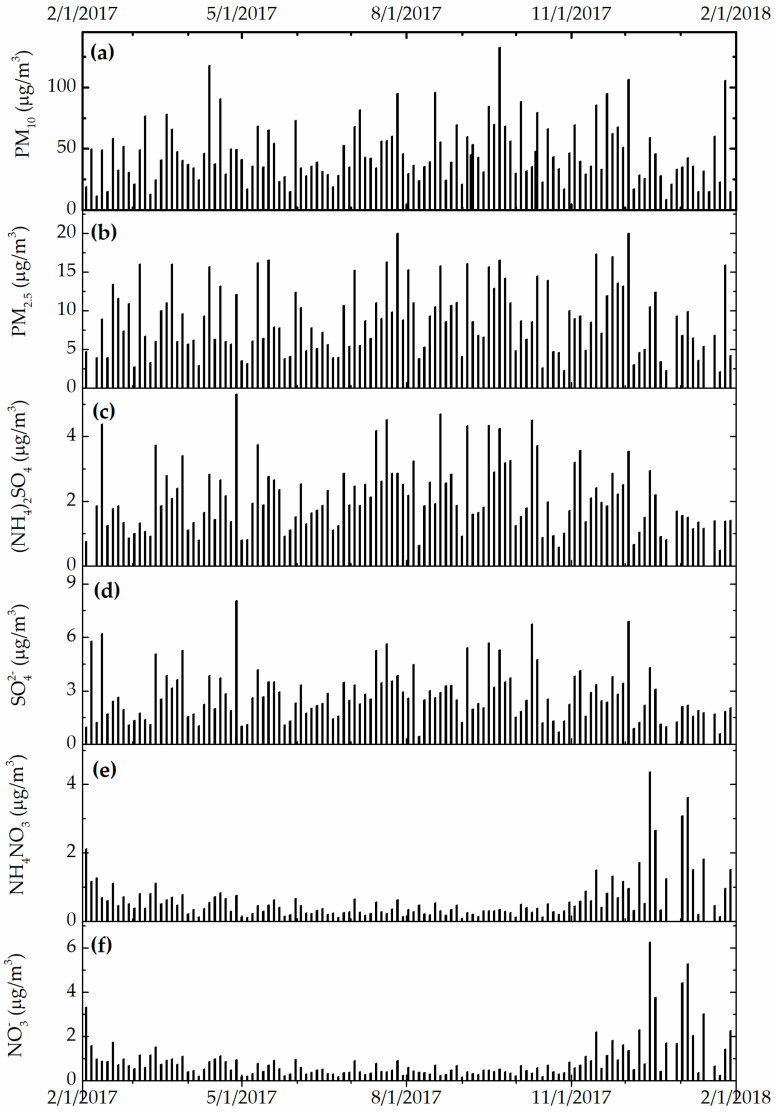
(**a**) Daily PM_10_, (**b**) PM_2.5_, (**c**) bisulfate, (**d**) nitrate, (**e**) total sulfate, and (**f**) total nitrate concentration (in μg/m^3^).

**Figure 5:**
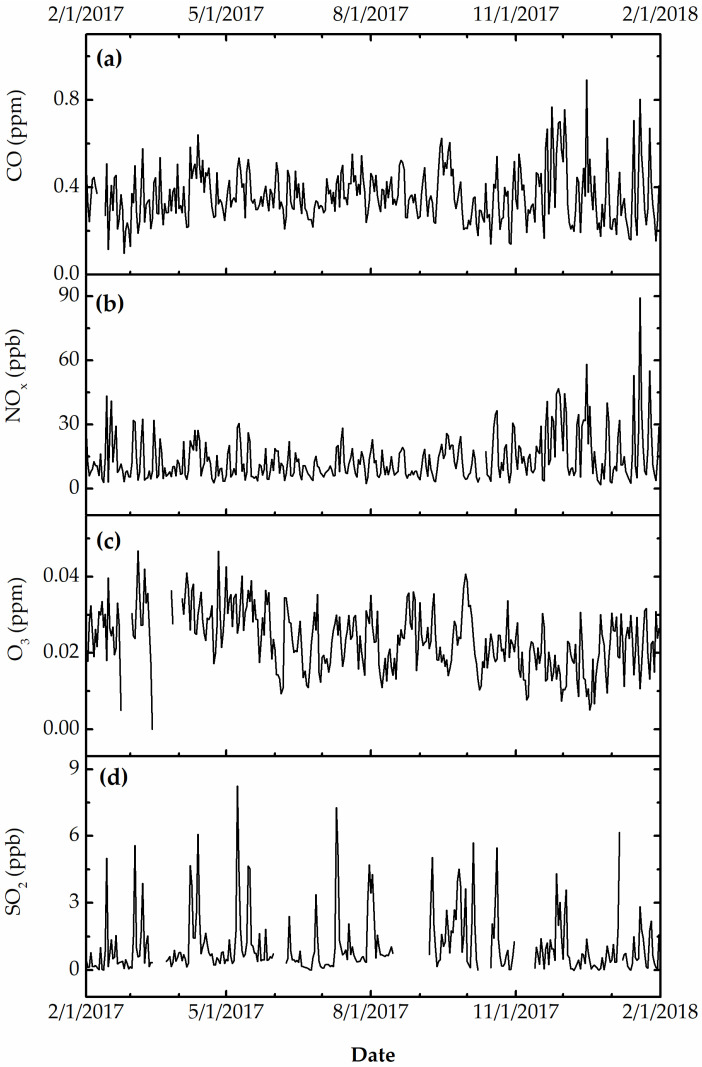
(**a**, in ppm) Daily carbon monoxide, (**b**, ppb) nitrogen oxides, (**c**, ppm) ozone, and (**d**, in ppb) sulfur dioxide.

Figure 3, Figure 4 and Figure 5 show the daily variation of meteorological, particulate, and gas pollutants with the study period, respectively. The mean daily temperature and relative humidity were 18.3 °F and 66%, respectively. There was a total of 123 rain events for a total of 619.3 cm of H_2_O. The winds were blowing from a variable direction, mostly across the southwest–northeast axis, up to 1.77 m/s on average. The mean daily ambient PM_10_ and PM_2.5_ mass concentrations were 45.8 and 8.8 μg/m^3^, indicating that a large fraction of ambient PM_10_ was composed of coarse particles. The total sulfate and nitrate concentrations (2.7 μg/m^3^ and 0.9 μg/m^3^) were substantially higher than those computed from neutralized ammonium bisulfate (2.09 μg/m^3^) and ammonium nitrate (0.6 μg/m^3^), suggesting acidic aerosol conditions that favored the deposition of sulfuric and nitric acid to surfaces. The mean daily CO, O_3_, NO_x_, and SO_2_ concentrations were 360 ppb, 23 ppb, 14 ppb, and 9 ppb, respectively. The variation of meteorological and air pollution conditions was typical of an urban environment. It is worth noting that the air quality monitoring site was in close proximity of traffic and other industrial emissions, yielding reduced O_3_ concentrations due to titration by combustion-related NO_x_.

**Figure 6:**
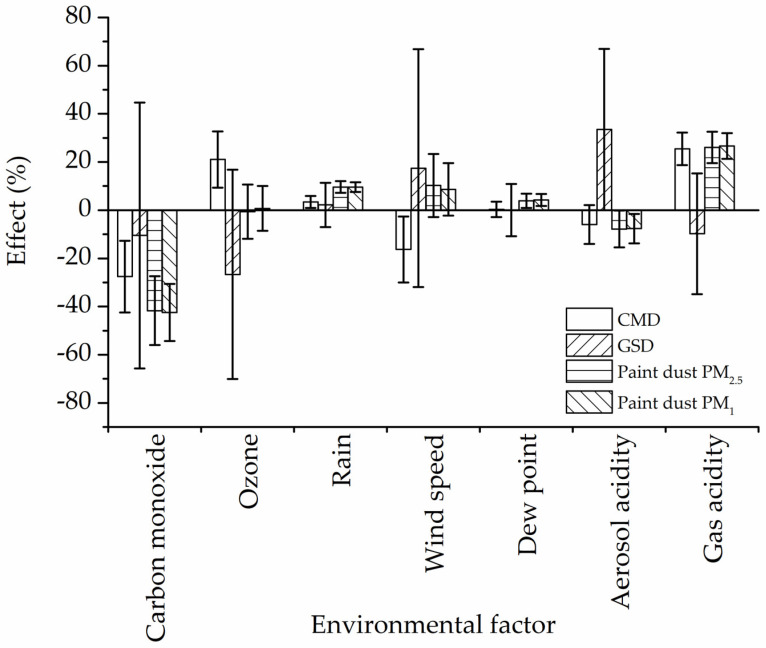
Effect (%) of environmental weathering conditions on paint dust PM_2.5_, PM_1_, CMD, and GSD.

Figure 6 shows the percent of relative effect of weathering factors on paint dust CMD, GSD, PM_2.5_ and PM_1_ mass concentrations. The overall effect of meteorological conditions (dew point, rain, and wind) was positive, i.e., more paint dust being generated by abrasion, probably due to physical decomposition of paint and wood. The negative relative effect of CO and aerosol acidity for CMD and GSD indicated that smaller particles may be generated and that, therefore, the resultant PM_2.5_ and PM_1_ paint dust mass would decline, as computed by the regression analysis. On the other hand, the exposure to strong gas oxidants such as O_3_, NO_x_, and SO_2_ may result in the generation of a larger particle that was associated with an increase of paint dust mass.

The degradation of painted surfaces exposed to weathering occurs at the surface of the paint, inside the paint layer, and at the interface between the film and the substrate. Weathering conditions degrade painted surfaces through chain-scission and cross-linking reactions in the paint layer, deteriorating adhesion and the mechanical compatibility with the substrate. Previous studies clearly demonstrated the capacity of oxidative agents (e.g., acid rain, ozone, and radicals) to inadvertently cause significant damage to painted surfaces [29]. However, there is no evidence of chemical interactions of paint formulations, TiO_2_ materials, and CO [30]. In an urban environment, CO is released predominantly from automobiles. Traffic exhaust is also the primary source of volatile organic compounds that can readily react in the atmosphere and form hydroxyperoxy radicals. Paints may undergo oxidation and isomerization from a non-conjugated to a conjugated isomer and form hydroperoxides at allylic and vinylic carbons. This was in agreement with changes in the FTIR spectra of paint dust. These hydroperoxides may form dimers and polymers to an insoluble and infusible film. These reactions may occur throughout the life of the paint but at decreasing rates.

Water also accelerates deterioration by stimulating physical processes and volatile and water-soluble decomposition byproducts. In conjunction with a high ambient temperature, which is typical in the southeast US, degradation reactions may accelerate depending on the reactions’ activation energy. These processes may be further facilitated by TiO_2_ particles. Anatase titanium dioxide participates chemically in the deterioration reaction by acting as a catalyst in the oxidation of film by atmospheric oxygen [2].

The discharge of TiO_2_ particles in the environment has been hypothesized to involve their release from the outer layer due to dissolution followed by diffusion into the solution in cement surfaces [31]. Environmental releases of TiO_2_ particles appear to be negligible during the production of paints [32], but they have been previously found in environmental media including ambient air [33,34].

## 4. Conclusions

The effect of environmental weathering on TiO_2_-containing paint dust emissions by mechanical abrasion was investigated. This study showed that most TiO_2_ and paint polymers may have been depleted from weathered painted surfaces; PM_10_, PM_2.5_, and PM_1_ paint dust emissions declined as a narrow range (GSD decrease) of smaller particles (CMD decrease) was generated for weathered wood panels. The morphological and chemical analysis showed that the abundance of TiO_2_ agglomerates in the nano-size range declined over time. Weathering agents including meteorological conditions and air pollutants may act at different levels and rates, independently or synergistically in various combinations and in different sequences, resulting in highly variable outcomes. Gas and aerosol acidity and rain appeared to exert a strong influence on paint deterioration. The strong association with carbon monoxide, an indicator of incomplete combustion, may be indicative of the potential effect of highly reactive volatile organic compounds, co-emitted with CO from vehicles. This study demonstrated the need to assess the synergistic effects of environmental weathering on painted surfaces because of its potential to expose the environment and workers to appreciable quantities of TiO_2_-containing paint dust.

## Figures and Tables

**Table 1:** Monthly paint dust CMD, GSD, and mass concentrations and trend (mean ± 3× standard error).

Duration (Month)	CMD (nm)	GSD	Particle Mass (μg/m^3^)		
			PM_10_	PM_2.5_	PM_1_
1	60 ± 22	3.4 ± 0.5	1250 ± 715	71 ± 43	18 ± 11
2	52 ± 12	3.9 ± 0.7	1573 ± 833	84 ± 39	22 ± 10
3	51 ± 14	2.9 ± 0.5	764 ± 471	38 ± 20	10 ± 5
4	77 ± 28	2.9 ± 0.5	1443 ± 1023	92 ± 68	25 ± 19
5	68 ± 18	2.8 ± 0.5	1887 ± 2325	130 ± 143	37 ± 8
6	62 ± 8	2.8 ± 0.2	832 ± 353	79 ± 36	24 ± 11
7	39 ± 10	2.5 ± 0.1	247 ± 166	25 ± 16	9 ± 6
8	50 ± 12	2.7 ± 0.2	315 ± 154	26 ± 17	8 ± 5
9	61 ± 12	2.5 ± 0.1	373 ± 416	38 ± 32	12 ± 8
10	69 ± 10	2.3 ± 0.1	315 ± 225	39 ± 22	13 ± 7
11	98 ± 29	2.4 ± 0.2	577 ± 380	64 ± 27	22 ± 7
12	82 ± 9	2.3 ± 0.1	118 ± 78	13 ± 9	4 ± 3
Trend	2.2 ± 1.2	−0.12 ± 0.02 (1/month)	−123 ± 35	−5 ± 3	−1 ± 0.8
	(nm/month)		(μg/m^3^/month)	(μg/m^3^/month)	(μg/m^3^/month)

## Data Availability

The data presented in this study are available on request from the corresponding author.
